# 2,2′-[Ethane-1,2-diylbis(sulfanedi­yl)]bis­(pyridine *N*-oxide)

**DOI:** 10.1107/S1600536809050788

**Published:** 2009-11-28

**Authors:** Huan-Huan Wang, Chao-Yan Zhang, Yue Cui, Ya-Bo Xie

**Affiliations:** a College of Environmental and Energy Engineering, Beijing University of Technology, Beijing 100124, People’s Republic of China

## Abstract

The tile compound, C_12_H_12_N_2_O_2_S_2_, lies on an inversion center. The two pyridyl rings are parallel to each other. The structure is devoid of any classical hydrogen bonds due to lack of appropriate donors and acceptors for such bonds. However, non-classical hydrogen bonds of the types C—H⋯O and C—H⋯S stabilize the structure.

## Related literature

For thio­ether-type complexes, see: Xie *et al.* (2006 ▶). For a related structure, see: Zhang *et al.* (2009 ▶).
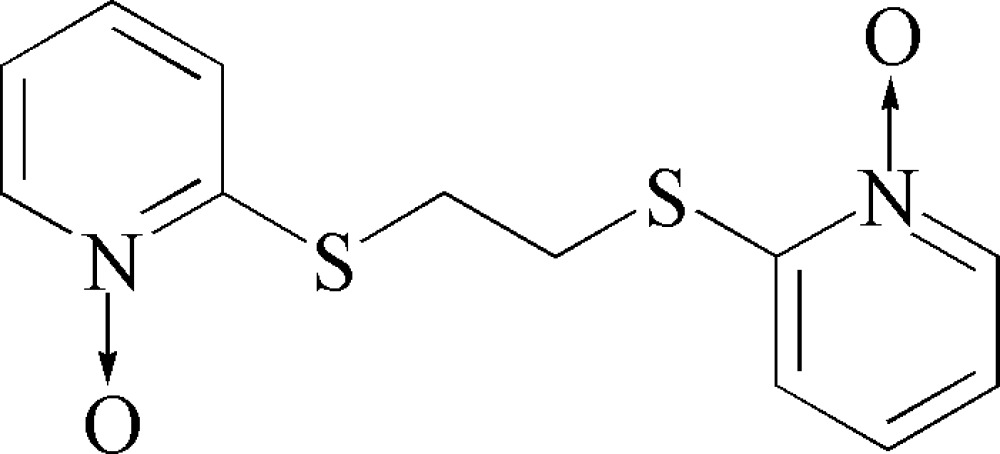


## Experimental

### 

#### Crystal data

C_12_H_12_N_2_O_2_S_2_
*M* *_r_* = 280.36
Monoclinic, 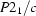

*a* = 8.2776 (17) Å
*b* = 6.9790 (14) Å
*c* = 10.791 (2) Å
β = 93.52 (3)°
*V* = 622.2 (2) Å^3^
*Z* = 2
Mo *K*α radiation
μ = 0.42 mm^−1^
*T* = 293 K
0.28 × 0.26 × 0.24 mm

#### Data collection

Bruker SMART CCD area-detector diffractometer
Absorption correction: multi-scan (*SADABS*; Bruker, 1998 ▶) *T*_min_ = 0.889, *T*_max_ = 0.904
3068 measured reflections
1098 independent reflections
1007 reflections with *I* > 2(*I*)
*R*_int_ = 0.013

#### Refinement

*R*[*F* ^2^ > 2σ(*F* ^2^)] = 0.025
*wR*(*F* ^2^) = 0.070
*S* = 1.07
1098 reflections
82 parameters
H-atom parameters constrained
Δρ_max_ = 0.14 e Å^−3^
Δρ_min_ = −0.22 e Å^−3^

### 

Data collection: *XSCANS* (Bruker, 1998 ▶); cell refinement: *XSCANS*; data reduction: *SHELXTL* (Sheldrick, 2008 ▶); program(s) used to solve structure: *SHELXS97* (Sheldrick, 2008 ▶); program(s) used to refine structure: *SHELXL97* (Sheldrick, 2008 ▶); molecular graphics: *SHELXTL*; software used to prepare material for publication: *SHELXTL*.

## Supplementary Material

Crystal structure: contains datablocks I, global. DOI: 10.1107/S1600536809050788/pv2239sup1.cif

Structure factors: contains datablocks I. DOI: 10.1107/S1600536809050788/pv2239Isup2.hkl

Additional supplementary materials:  crystallographic information; 3D view; checkCIF report

## Figures and Tables

**Table 1:** Hydrogen-bond geometry (Å, °)

*D*—H⋯*A*	*D*—H	H⋯*A*	*D*⋯*A*	*D*—H⋯*A*
C1—H1*A*⋯O1^i^	0.96	2.30	3.225 (2)	161
C4—H4*A*⋯S1^ii^	0.96	2.85	3.599 (2)	135

Symmetry codes: (i) 
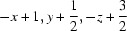
; (ii) 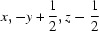.

**Fig. 1.**
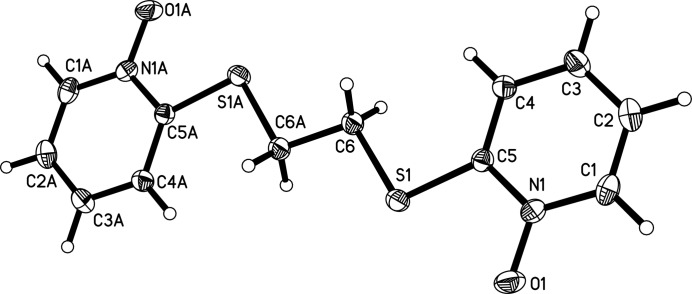
The molecular structure of the title compound with displacement ellipsoids drawn at the 30% probability level for non-hydrogen atoms.

## supplementary crystallographic information

### Comment

In the past decades, there were many reports about the thioether-type compounds with their flexibility and conformation freedoms (Xie *et al.*, 2006). As a continuation of our series of research on thioether-type compounds (Zhang *et al.*, 2009), we report herein the crystal structure of the title compound.

The title compound (Fig.1) was obtained by the reaction of 2-mercaptopyridine N-oxide and 1,2-dibromoethane. There exits a symmetrical center located at the midpoint of the two methylenes and the pyridyl rings of the title compound are parallel to each other. Thestructure is stabilized by non-classical hydrogen bonds of the types C—H···O and C—H···S.

### Experimental

2-Mercaptopyridine N-oxide (1.2719 g, 10.0 mmol) was added to a stirred and heated solution of KOH (0.5837 g, 10.4 mmol) in ethanol (50 ml). After 30 min, 1,2-dibromoethane (0.9917 g, 5.3 mmol) was added and reacted for 10 h. The mixture was cooled to room temperature and the precipitate was filtered off and washed with water, giving a white powder. After slow diffusion of ether into the solution of the powder in CHCl_3_/CH_3_CH_2_OH, colorless block single crystals suitable for X-ray diffraction were collected.

### Refinement

All H atoms were included at geometrically idealized positions with C—H = 0.96 Å and treated as riding with *U*_iso_(H) = 1.2 and 1.5 *U*_eq_(C_aryl_ and C_methylene_, respectively).

### Crystal data

**Table:**

C_12_H_12_N_2_O_2_S_2_	*F*(000) = 292
*M**_r_* = 280.36	*D*_x_ = 1.496 Mg m^−^^3^
Monoclinic, *P*2_1_/*c*	Mo *K*α radiation, λ = 0.71073 Å
Hall symbol: -P 2ybc	Cell parameters from 3720 reflections
*a* = 8.2776 (17) Å	θ = 2.5–27.9°
*b* = 6.9790 (14) Å	µ = 0.42 mm^−^^1^
*c* = 10.791 (2) Å	*T* = 293 K
β = 93.52 (3)°	Block, colorless
*V* = 622.2 (2) Å^3^	0.28 × 0.26 × 0.24 mm
*Z* = 2	

### Data collection

**Table:**

Bruker SMART CCD area-detector diffractometer	1098 independent reflections
Radiation source: fine-focus sealed tube	1007 reflections with *I* > 2(*I*)
graphite	*R*_int_ = 0.013
ω scans	θ_max_ = 25.0°, θ_min_ = 2.5°
Absorption correction: multi-scan (*SADABS*; Bruker, 1998)	*h* = −9→7
*T*_min_ = 0.889, *T*_max_ = 0.904	*k* = −8→8
3068 measured reflections	*l* = −12→12

### Refinement

**Table:**

Refinement on *F*^2^	Primary atom site location: structure-invariant direct methods
Least-squares matrix: full	Secondary atom site location: difference Fourier map
*R*[*F*^2^ > 2σ(*F*^2^)] = 0.025	Hydrogen site location: inferred from neighbouring sites
*wR*(*F*^2^) = 0.070	H-atom parameters constrained
*S* = 1.07	*w* = 1/[σ^2^(*F*_o_^2^) + (0.0387*P*)^2^ + 0.1633*P*] where *P* = (*F*_o_^2^ + 2*F*_c_^2^)/3
1098 reflections	(Δ/σ)_max_ = 0.006
82 parameters	Δρ_max_ = 0.14 e Å^−^^3^
0 restraints	Δρ_min_ = −0.22 e Å^−^^3^

### Special details

**Table:**

Geometry. All e.s.d.'s (except the e.s.d. in the dihedral angle between two l.s. planes) are estimated using the full covariance matrix. The cell e.s.d.'s are taken into account individually in the estimation of e.s.d.'s in distances, angles and torsion angles; correlations between e.s.d.'s in cell parameters are only used when they are defined by crystal symmetry. An approximate (isotropic) treatment of cell e.s.d.'s is used for estimating e.s.d.'s involving l.s. planes.
Refinement. Refinement of *F*^2^ against ALL reflections. The weighted *R*-factor *wR* and goodness of fit *S* are based on *F*^2^, conventional *R*-factors *R* are based on *F*, with *F* set to zero for negative *F*^2^. The threshold expression of *F*^2^ > σ(*F*^2^) is used only for calculating *R*-factors(gt) *etc*. and is not relevant to the choice of reflections for refinement. *R*-factors based on *F*^2^ are statistically about twice as large as those based on *F*, and *R*- factors based on ALL data will be even larger.

### Fractional atomic coordinates and isotropic or equivalent isotropic displacement parameters (Å^2^)

**Table:**

	*x*	*y*	*z*	*U*_iso_*/*U*_eq_	
S1	0.15370 (5)	0.18793 (5)	0.61401 (3)	0.03644 (16)	
N1	0.32129 (13)	0.50095 (17)	0.63713 (10)	0.0304 (3)	
O1	0.33935 (13)	0.43441 (16)	0.75096 (9)	0.0434 (3)	
C5	0.22742 (15)	0.39913 (19)	0.55220 (12)	0.0285 (3)	
C6	0.04101 (17)	0.0906 (2)	0.47863 (13)	0.0336 (3)	
H7B	−0.0423	0.1793	0.4512	0.050*	
H7C	0.1130	0.0708	0.4134	0.050*	
C1	0.39499 (18)	0.6652 (2)	0.60369 (16)	0.0377 (4)	
H1A	0.4632	0.7341	0.6635	0.045*	
C4	0.20326 (17)	0.4676 (2)	0.43202 (13)	0.0353 (3)	
H4A	0.1378	0.3962	0.3718	0.042*	
C2	0.37221 (18)	0.7344 (2)	0.48487 (15)	0.0417 (4)	
H6A	0.4235	0.8516	0.4622	0.050*	
C3	0.27461 (19)	0.6363 (2)	0.39827 (15)	0.0415 (4)	
H5A	0.2573	0.6851	0.3153	0.050*	

### Atomic displacement parameters (Å^2^)

**Table:**

	*U*^11^	*U*^22^	*U*^33^	*U*^12^	*U*^13^	*U*^23^
S1	0.0473 (3)	0.0327 (2)	0.0285 (2)	−0.00976 (16)	−0.00380 (16)	0.00374 (14)
N1	0.0298 (6)	0.0312 (6)	0.0300 (6)	0.0024 (5)	0.0001 (5)	−0.0051 (5)
O1	0.0515 (6)	0.0479 (7)	0.0295 (6)	−0.0003 (5)	−0.0092 (5)	−0.0024 (5)
C5	0.0278 (7)	0.0285 (7)	0.0292 (7)	−0.0003 (5)	0.0019 (5)	−0.0023 (6)
C6	0.0378 (8)	0.0320 (8)	0.0308 (7)	−0.0061 (6)	0.0008 (6)	−0.0004 (6)
C1	0.0319 (7)	0.0320 (7)	0.0494 (9)	−0.0040 (6)	0.0038 (6)	−0.0119 (7)
C4	0.0386 (8)	0.0382 (8)	0.0290 (7)	−0.0056 (6)	0.0011 (6)	0.0003 (6)
C2	0.0412 (9)	0.0325 (8)	0.0528 (10)	−0.0052 (7)	0.0132 (7)	−0.0007 (7)
C3	0.0469 (9)	0.0408 (8)	0.0375 (8)	−0.0029 (7)	0.0081 (7)	0.0068 (7)

### Geometric parameters (Å, °)

**Table:**

S1—C5	1.7436 (14)	C6—H7C	0.9600
S1—C6	1.8160 (15)	C1—C2	1.372 (2)
N1—O1	1.3131 (16)	C1—H1A	0.9600
N1—C1	1.3578 (19)	C4—C3	1.377 (2)
N1—C5	1.3642 (18)	C4—H4A	0.9600
C5—C4	1.385 (2)	C2—C3	1.379 (2)
C6—C6^i^	1.521 (3)	C2—H6A	0.9601
C6—H7B	0.9600	C3—H5A	0.9601
			
C5—S1—C6	100.54 (6)	N1—C1—C2	120.48 (14)
O1—N1—C1	121.33 (12)	N1—C1—H1A	120.0
O1—N1—C5	118.14 (12)	C2—C1—H1A	119.5
C1—N1—C5	120.52 (12)	C3—C4—C5	120.21 (14)
N1—C5—C4	119.53 (13)	C3—C4—H4A	119.9
N1—C5—S1	112.44 (10)	C5—C4—H4A	119.9
C4—C5—S1	128.02 (11)	C1—C2—C3	119.98 (15)
C6^i^—C6—S1	106.50 (13)	C1—C2—H6A	120.0
C6^i^—C6—H7B	107.7	C3—C2—H6A	120.0
S1—C6—H7B	109.3	C4—C3—C2	119.23 (15)
C6^i^—C6—H7C	114.3	C4—C3—H5A	120.5
S1—C6—H7C	109.4	C2—C3—H5A	120.3
H7B—C6—H7C	109.5		
			
O1—N1—C5—C4	178.66 (12)	O1—N1—C1—C2	−178.76 (13)
C1—N1—C5—C4	−2.06 (19)	C5—N1—C1—C2	2.0 (2)
O1—N1—C5—S1	−2.27 (15)	N1—C5—C4—C3	0.6 (2)
C1—N1—C5—S1	177.01 (10)	S1—C5—C4—C3	−178.35 (12)
C6—S1—C5—N1	−178.99 (10)	N1—C1—C2—C3	−0.4 (2)
C6—S1—C5—C4	−0.02 (15)	C5—C4—C3—C2	1.0 (2)
C5—S1—C6—C6^i^	−176.25 (13)	C1—C2—C3—C4	−1.1 (2)

Symmetry codes: (i) −*x*, −*y*, −*z*+1.

### Hydrogen-bond geometry (Å, °)

**Table:**

*D*—H···*A*	*D*—H	H···*A*	*D*···*A*	*D*—H···*A*
C1—H1A···O1^ii^	0.96	2.30	3.225 (2)	161
C4—H4A···S1^iii^	0.96	2.85	3.599 (2)	135

Symmetry codes: (ii) −*x*+1, *y*+1/2, −*z*+3/2; (iii) *x*, −*y*+1/2, *z*−1/2.
